# Lewy body disease as a potential negative outcome modifier of glioblastoma treatment: a case report

**DOI:** 10.1186/s12883-023-03313-4

**Published:** 2023-07-04

**Authors:** Eric T. Wong, Harry Rosenberg, Olivia Dawood, Lauren Hertan, Rafael A. Vega, Matthew Anderson, Erik J. Uhlmann

**Affiliations:** 1 Brain Tumor Center & Neuro-Oncology Unit, Department of Neurology, Beth Israel Deaconess Medical Center, 330 Brookline Ave, Boston, 02215 United States; 2 Department of Neurology, Medicine (Division of Hematology/Oncology), Neurosurgery & Radiation Oncology, Rhode Island Hospital, 593 Eddy St, Providence, 02903 United States; 3 Division of Neuropathology, Department of Pathology, Beth Israel Deaconess Medical Center, 330 Brookline Ave, Boston, 02215 United States; 4 Department of Radiation Oncology, Beth Israel Deaconess Medical Center, 330 Brookline Ave, Boston, 02215 United States; 5 Division of Neurosurgery, Department of Surgery, Beth Israel Deaconess Medical Center, 330 Brookline Ave, Boston, 02215 United States; 6 Present Address: Regeneron Pharmaceutical Company, 777 Old Saw Mill Rive Road, Tarrytown, NY 10591 United States

**Keywords:** Glioblastoma, Lewy Bodies, Dementia, Outcome Modifier

## Abstract

**Background:**

Elderly patients with glioblastoma are particularly susceptible to the adverse effects of ionizing radiation to the brain. This population also has an increasing prevalence of dementia in the successive seventh, eighth and nineth decade of life, and dementia with Lewy bodies is characterized by pathologic α-synucleins, proteins that take part in neuronal DNA damage repair.

**Case presentation:**

We report a 77-year-old man, with a history of coronary artery disease and mild cognitive impairment, who experienced subacute behavioral changes over 3 months with wording-finding difficulty, memory loss, confusion, perseveration, and irritable mood. Neuroimaging studies disclosed a 2.5 × 2.4 × 2.7 cm cystic enhancing mass with central necrosis in the left temporal lobe of the brain. Gross total resection of the tumor revealed IDH-1 wild-type glioblastoma. After treatment with radiation and temozolomide chemotherapy, his cognitive status deteriorated rapidly, and he died from unexpected sudden death 2 months after radiation. Autopsy of his brain revealed (i) tumor cells with atypical nuclei and small lymphocytes, (ii) neuronal cytoplasmic inclusions and Lewy bodies that were positive for α-synuclein in the midbrain, pons, amygdala, putamen and globus pallidus, and (iii) no amyloid plaques and only rare neurofibrillary tangles near the hippocampi.

**Conclusions:**

This patient most likely had pre-clinical limbic subtype of dementia with Lewy bodies prior to his diagnosis of glioblastoma. The radiation and temozolomide that was used to treat his tumor may have accelerated neuronal damage due to induction of DNA breakage when his brain was already compromised by pathologic α-synucleins. α-Synucleinopathy could be a negative outcome modifier in glioblastoma patients.

## Background

The overall incidence of glioblastoma is approximately 13 per 100,000 persons, and the age-adjusted incidence increases in successive decades after age 60 [1]. Elderly patients in particular have a poor prognosis despite treatment. The standard Stupp protocol, which consists of radiotherapy delivered over 6 weeks together with concomitant daily temozolomide followed by adjuvant monthly temozolomide, included only patients 70 years of age or younger [2]. Although a post hoc analysis of the trial cohorts between the age of 65 and 70 did not demonstrate a benefit from the addition of temozolomide [3], the combination of hypofractionated radiotherapy and temozolomide over 3 weeks still showed improved survival in this population when investigated in a randomized prospective phase 3 trial [4]. Finding from these studies suggests that the elderly derives only a small benefit from temozolomide treatment and hypofractionated radiotherapy offers the optimal balance between efficacy and toxicity. However, toxicity from hypofractionated radiotherapy or conventionally fractionated radiation on neurological functions in this population is still poorly understood.

Older patients are particularly susceptible to both systemic and neurological side effects of chemotherapy and radiation treatments. Population studies have shown that over 60% of the individuals at age 65 or older have 2 or more systemic co-morbidities, including concurrent heart disease, chronic obstructive pulmonary disease, diabetes, kidney disease and stroke [5]. Furthermore, the prevalence of dementia also increases in these same patients. Alzheimer’s disease and vascular dementia represent the most common and second most common type of memory disorders, respectively, occurring at a combined rate of 5% in the seventh, 25% in the eighth and 37% in the ninth decade [6]. Lewy body dementia is third most common and comprises 5% of all dementia cases [7, 8]. Although the overall incidence is < 10 per 100,000 person-years, this incidence increases significantly in each successive decades after age 60 [9, 10]. Here, we report a patient who underwent an uncomplicated gross total resection of a left temporal lobe glioblastoma followed by adjuvant treatment using the Stupp protocol; however, he experienced a rapid neurocognitive deterioration shortly after completion of radiation. This patient died suddenly, and a post-mortem examination of the brain revealed the presence of Lewy bodies in multiple subcortical regions.

## Case presentation

**Fig. 1:**
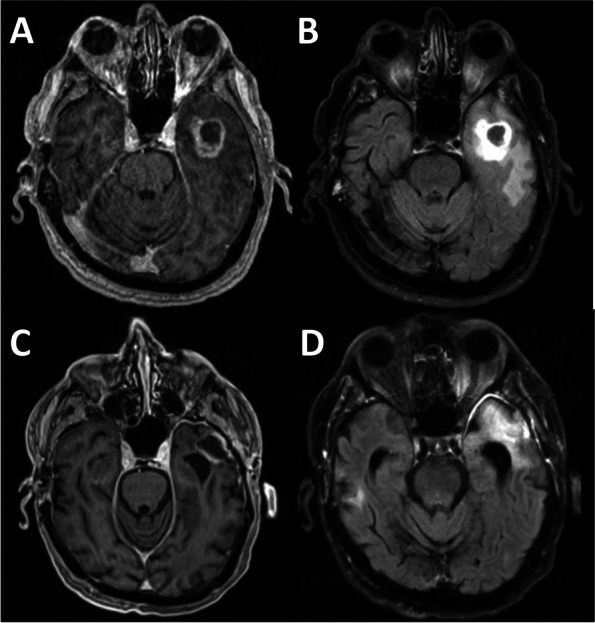
IDH-1 wild-type glioblastoma before and after surgery, radiation, and chemotherapy. MRI from post-gadolinium T1-weighted MP-RAGE (**A**) and FLAIR (**B**) sequences demonstrating irregular cystic enhancement and adjacent cerebral edema. The surgical cavity after gross total resection and external beam involved-field radiation as seen on the post-gadolinium T1-weighted MP-RAGE (**C**) and FLAIR (**D**) sequences

The patient was a 77-year-old right-handed Caucasian man who developed confusion, word-finding difficulty, and slurred speech in early 2020. These symptoms include making phone calls to his wife and asking her non-sensible questions. He lost his words intermittently and forgot names of his family members on several occasions. He recounted old stories as if he were perseverating and answered questions in the affirmative without hearing the question. His mood also became more irritable. His past medical history was notable for a skull fracture after a fall from horseback riding when he was a teenager. As a result, he had undergone a neurocognitive evaluation at age 75 from a memory disorder clinic in a major academic medical center in 2017, and he was diagnosed with a non-specific memory disorder that did not affect his activities of daily living. A non-contrast head CT, obtained in July 2020 after 3 months into his progressive symptoms, showed a hypodense mass in the left temporal lobe of the brain. On the following day, a CT of the torso was unremarkable, but a gadolinium-enhanced head MRI revealed a cystic enhancing mass with central necrosis, measuring 2.5 × 2.4 × 2.7 cm, (Fig. 1A) together with mild cerebral atrophy globally. The mass was associated with extensive T2 and FLAIR (Fig. 1B) signals extending from the inferior left temporal lobe to the insular cortex as well as the internal capsule and the peri-ventricular white matter near the occipital horn on the left side. There was a 6 mm rightward midline shift together with mild effacement of the ambient cistern. The patient underwent a gross total resection of the mass without complication 4 days after obtaining the MRI. Pathology revealed glioblastoma with histological features notable for dense cellularity, moderate to severe atypia and easily identified mitoses. Immunohistochemical staining was negative for the IDH-1 R132H mutation but positive for ATRX, GFAP, Olig-2 and p53. The Ki-67 index showed 20–25% positivity indicating a moderate proliferation rate. Methylation study for the promoter of O^6^-methylguanine-DNA methyltransferase was not performed. Three weeks after surgery, the patient started involved-field radiotherapy and concomitant daily temozolomide, but only 2 weeks of chemotherapy was administered due to thrombocytopenia, and 6 weeks of radiation treatment was eventually completed 2 months after diagnosis (Fig. 1C and D).

**Fig. 2:**
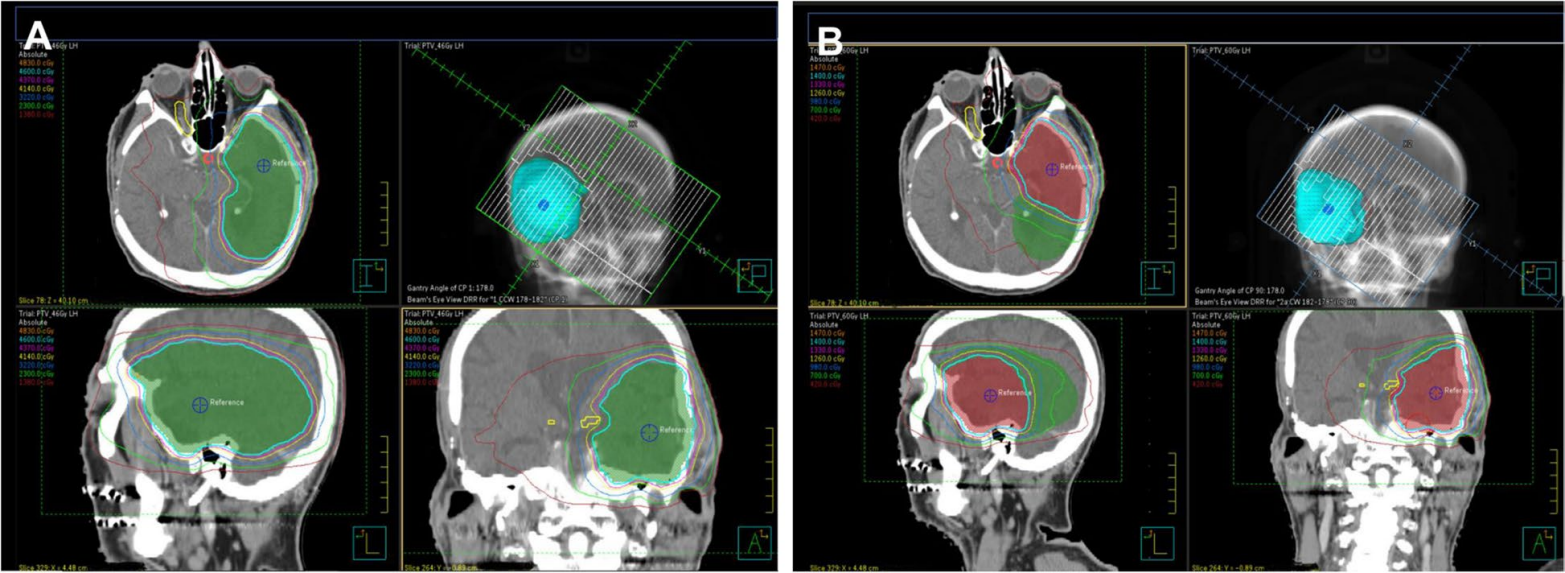
Radiation mapping diagrams for VMAT. The clinical tumor volume, which consists of the surgical cavity after gross total resection, FLAIR positive region and a margin of 2 cm, received 4,600 cGy in 200 cGy per fraction over 23 days (**A**). This is followed by a boost to the surgical cavity and FLAIR positive region to 1,400 cGy also in 200 cGy per fraction for an additional 7 days (**B**)

Standard external beam radiotherapy was delivered using volumetric modulated arc therapy (VMAT) to a total dose of 6,000 cGy over 30 fractions or 200 cGy per fraction delivered in 2 courses. Target volumes were delineated on a planning CT, which were anatomically co-registered with MP-RAGE and FLAIR MRI images. The first course of radiation consisted of 4,600 cGy delivered over 23 fractions to the surgical cavity, gadolinium-enhancing tumor and FLAIR hyperintensity, plus a margin of 2 cm cropped to anatomic barriers with an additional 3 mm planning margin (target volume named PTV_46Gy) (Fig. 2A). The second course or boost was delivered sequentially, with an additional 1,400 cGy in 7 fractions to the surgical cavity plus gadolinium enhancement and a margin of 2 cm cropped to anatomic barriers with an additional 3 mm planning margin (target volume named PTV_60Gy) (Fig. 2B). Over 96% of the PTV_46Gy received the prescription dose of 4,600 cGy in the first treatment course, and over 95% of the PTV_60Gy received 6,000 cGy in the second. The left temporal postsurgical cavity as well as the ipsilateral putamen and globus pallidus were included in the maximum 6,000 cGy isodose line. The brainstem was covered in the 3,000 cGy isodose line while the contralateral putamen and globus pallidus received up to 1,800 cGy of radiation.

His cognitive status rapidly deteriorated after radiation and temozolomide. He became more forgetful and developed imbalance. He required 24-h supervision for his daily activities. A trial of dexamethasone did not help. Lumbar puncture was performed 4 months after glioblastoma diagnosis and showed 1 white blood cell/μL, 1 red blood cell/μL, 50 mg/dL protein, 66 mg/dL glucose, 18 IU/L lactate dehydrogenase, negative VDRL, no detectable immunoglobulin on immunofixation, negative PCR for HSV-1, HSV-2 and VZV, and negative cytology for malignant cells. Phospho-tau was not elevated at 29.6 pg/mL (normal < 54 pg/mL).

**Fig. 3:**
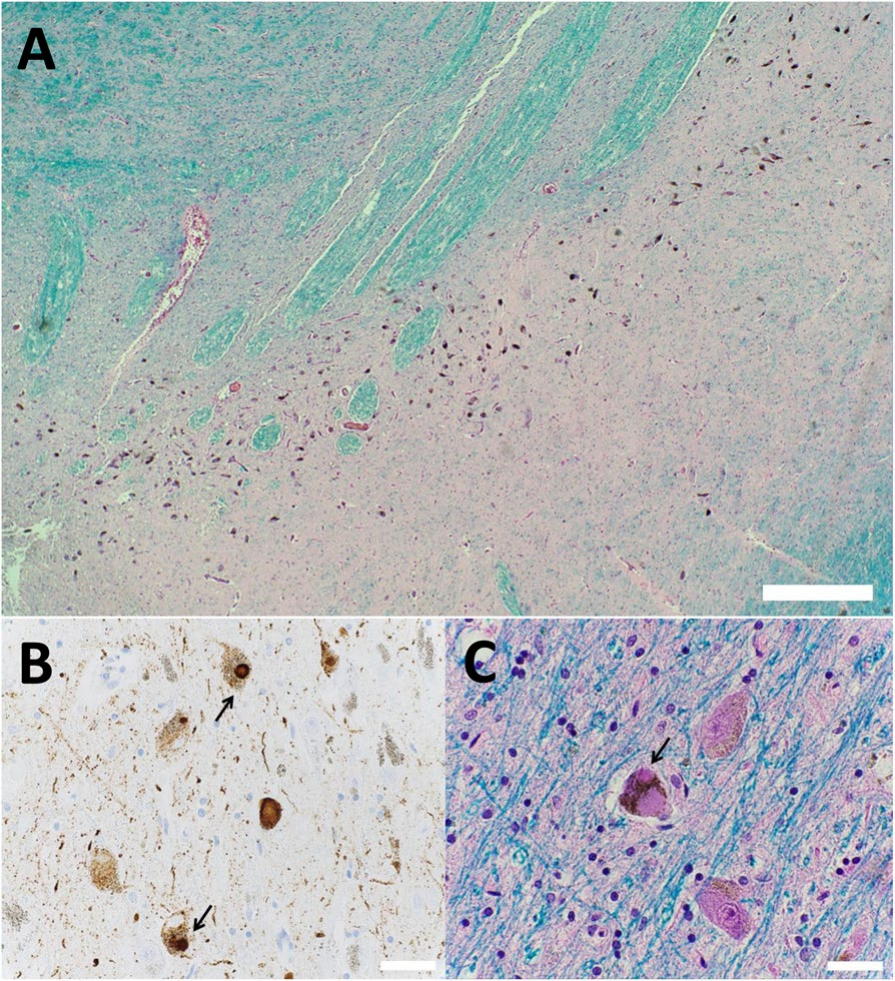
Autopsy findings in the brainstem. The brainstem showed a slight loss of myelination due to the radiation (**A**, scale bar = 200 μm). There is positive immunohistochemical staining for neuronal cytoplasmic α-synuclein (**B**, arrows, scale bar = 20 μm) and Lewy body (**C**, arrow, scale bar = 20 μm)

**Fig. 4:**
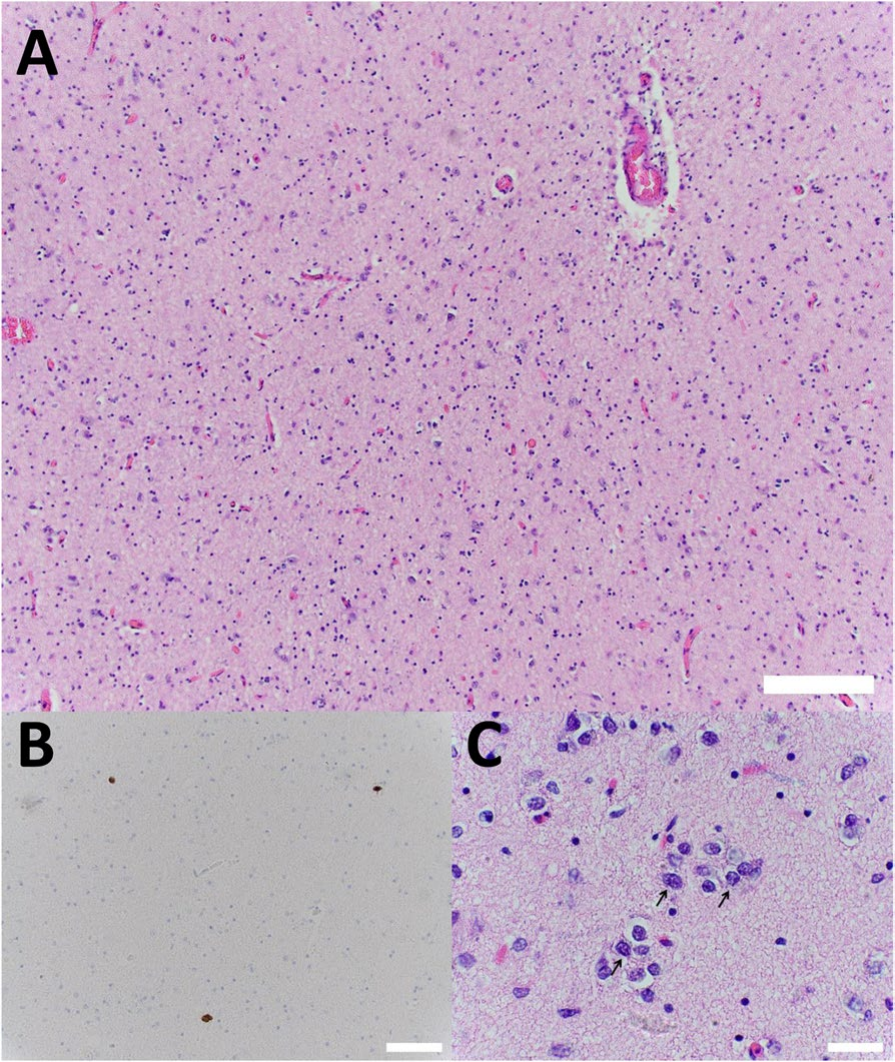
Hematoxylin and eosin staining of the left temporal pole. Lymphocytes were seen in the brain parenchyma (**A**, scale bar = 100 μm). The Ki-67 proliferation index was low (**B**, scale bar = 50 μm) and there were observable atypical tumor nuclei (**C**, arrows, scale bar = 20 μm)

He died unexpectedly from sudden death 2 months after completion of radiation or 4 months after establishment of his glioblastoma diagnosis. Post-mortem examination of the body revealed atherosclerosis of multiple coronary arteries, including (i) 25–50% narrowing of left main artery, (ii) 25–50% narrowing of the left anterior descending artery, and (iii) focal 75% narrowing of the right coronary arteries with a proximal stent. His lungs had emphysematous changes, but there was no evidence of pulmonary thromboembolism. His brain autopsy revealed slight loss of myelin in the brainstem (Figure 3A). Both neuronal cytoplasmic inclusions (Fig. 3B) and Lewy bodies (Fig. 3C) immunohistochemically stained positive for α-synuclein were identified in the midbrain, pons, amygdala, putamen and globus pallidus. At the left temporal pole, there were small lymphocytes (Fig. 4A), tumors cells with atypical nuclei and a low Ki-67 proliferation index (Fig. 4B and C), but no amyloid plaques and only rare neurofibrillary tangles in the cortex adjacent to the hippocampi.

## Discussion and conclusions

Our patient’s rapid neurocognitive decline after radiation led to speculation on the possibility that he had an intrinsic vulnerability to radiotherapy. The differential diagnosis of this rapid decline included communicating hydrocephalus, subacute encephalopathy, or subclinical neurodegenerative disease of the brain that was accelerated by radiation. A lumbar puncture showed normal cerebrospinal fluid pressure and his neurocognitive dysfunction did not improve after the procedure. Furthermore, phospho-tau level was low in the cerebrospinal fluid indicating either no tauopathy or at least a low burden of disease in the brain. Although the exact pathophysiology of subacute radiation-induced encephalopathy is unknown, this condition is usually reversible when given enough time and corticosteroid usually accelerates patient recovery. However, this patient did not show neurocognitive improvement despite treatment with dexamethasone. Therefore, a post-mortem examination of his brain was performed, which revealed widespread distribution of Lewy bodies in the subcortical regions while sparing the neocortex. Specifically, the presence of α-synuclein-positive Lewy bodies in the amygdala and basal ganglia is most consistent with the limbic (or transitional) subtype of dementia with Lewy bodies according to the latest Lewy Pathology Consensus Criteria [11].

Lewy bodies are found in patients with Parkinson’s disease, dementia with Lewy bodies, and multisystem atrophy [12]. They are intra-neuronal aggregates of α-synuclein fibrils phosphorylated at the serine 129 residues [13, 14]. Misfolded or overexpressed cytoplasmic α-synucleins can damage mitochondria and make neurons susceptible to oxidative stress [14, 15]. It is notable that ionizing radiation can also cause persistent oxidative stress within the irradiated tissue by damaging the mitochondria and generating reactive oxygen species, in the form of oxygen and hydroxyl radicals as well as hydrogen peroxide [16–18]. Our patient underwent involved-field radiotherapy for his left temporal glioblastoma and the irradiated hippocampus, putamen and globus pallidus on the left received up to 6,000 cGy of radiation while the right encountered a significantly lesser amount at 1,800 cGy or lower. Despite treatment for his tumor, our patient experienced an unusual and rapid decline in his neurocognitive function shortly after radiation, and post-mortem examination revealed Lewy bodies in the subcortical regions of his brain. In addition, we identified another patient who developed hemi-Parkinsonism 20 years after stereotactic radiosurgery to the right temporal lobe for metastatic melanoma, had decreased dopamine level in the ipsilateral striatum on Dopamine Transporter scan (DaTscan), and confirmed α-synuclein aggregates in peripheral nerve endings [15]. Together, external beam radiotherapy might have potentiated α-synuclein-induced neuronal injury, likely by acute and chronic oxidative stress.

Survey of the literature using PubMed revealed a number of cases with co-occurring Lewy body pathology and gliomas, including 2 anaplastic astrocytomas, 1 glioblastoma, and 1 gliomatosis cerebri (Table 1). First, these patients are older with a median age of 72 (range 61–77). Negative immunohistochemical staining for IDH-1 mutation was reported in 2, consistent with the typical profile in this older population [19]. Second, most of the cases were found to have Lewy body pathology at the brainstem, inferior frontal lobes and temporal lobes similar to our patient. Lastly, two had external beam radiation therapy, one received 4,000 cGy ionizing radiation to the right temporal lobe after biopsy [20]) and the other had the equivalent of 5,300 cGy from boron-neutron capture heavy particle therapy to the right parietal brain [21]. Only the latter was Lewy body pathology identified in the brainstem post-mortem, and the authors concluded that boron-neutron therapy was safe because this region only received an equivalent of 1,700 cGy of radiation [21]. In contrast, our patient received a much higher dose or 3,000 cGy to the brainstem, and therefore his neurologic vulnerability from Lewy bodies and the higher radiation dose could contribute in a synergistic fashion to his rapid deterioration after treatment.

**Table 1:** Survey of the literature using search terms (i) Lewy body dementia or Lewy bodies, and (ii) astrocytoma, oligodendroglioma, glioma, glioblastoma and gliomatosis

Articles	Year of Publication	Age	Gender	Type of Brain Tumor	Location(s) of Brain Tumo	IDH-1 Status	Location(s) of Lewy Bodies	Co-Morbid CNS Disease	Radiation	Chemotherapy	Survival (months)
Case Record of MGH [20]	1998	74	Male	Anaplastic astrocytoma	Right Temporal	N/A	Inferior frontal	─	4000 cGy	─	1 +
Oliveira HSD, et al. [22]	2018	77	Male	Gliomatosis cerebri	Right temporal, bilateral basal ganglia & bi-frontal	Wild-type	N/A	─	─	─	N/A
Aziz T, et al. [21]	2000	61	Male	Glioblastoma	Right Parietal	N/A	Midbrain & pons	─	5300 cGy	─	7
Leahy CB, et al. [23]	2022	70	Male	Anaplastic astrocytoma	Bilateral cerebral and cerebellar hemispheres, basal ganglia, midbrain, pons & medulla	Wild-type	Temporal lobe, midbrain, pons & medulla	Autonomic failure	─	─	84
Search Terms											
Lewy body dementia	and	Astrocytoma									
Lewy bodies		Oligodendroglioma									
		Glioma									
		Glioblastoma									
		Gliomatosis									

α-Synuclein is localized in the pre-synaptic region and its major function is to facilitate neuronal signaling [12]. It is also found in the nucleus and, in animal models, nuclear α-synuclein co-localizes with DNA damage response proteins ATM, γH2Ax and 53BP1, which are important for the repair of single- and double-strand breaks [16]. In addition, α-synuclein binds to double-stranded DNA damaged by the radiomimetic bleomycin and it may play a role with the Ku70/Ku80 complex in non-homologous end-joining repair [17, 18]. Furthermore, upon single-strand DNA damage, there is a 25-fold increase in the development of neurotoxic aggregates of α-synuclein and poly-ADP-ribose, the latter of which is generated by activated poly-ADP-ribose polymerase (PARP) [24]. Importantly, high levels of Lewy bodies are often found in the amygdala and the extent of pathology in patients with Lewy body disease correlates with the level of DNA double-strand breaks [17]. Since radiation triggers double-strand DNA breaks and temozolomide causes N-alkylation of purines requiring base-excision repair by PARP [18], glioblastoma patients with a high burden of α-synucleinopathy or Lewy bodies may be particularly susceptible to neuronal damage induced by concomitant radiation and temozolomide.

Elderly individuals often develop neurocognitive dysfunction after radiotherapy. Delayed encephalopathy or dementia is a dreaded irreversible complication of whole brain radiotherapy. This is thought to be a result of hippocampal damage, a site of continued neural genesis during adulthood. Indeed, bilateral hippocampal-sparing whole brain radiation with memantine preserves neurocognitive function better than the traditional fractionated whole brain radiotherapy [25]. Still, there is heterogeneity in the outcome of these patients and sparing of the hippocampi may not be enough to protect elderly patients. This is because the majority of neurodegenerative diseases have misfolded proteins that are localized to the nucleus and play a role in neuronal DNA repair in regions of the brain other than the hippocampi, including tau in Alzheimer’s disease, TDP-43 and FUS in frontotemporal lobar dementia and amyotrophic lateral sclerosis [26, 27], Trf2 in neurodegeneration from welding fume exposure [28, 29] and α-synuclein in Lewy body disease, Parkinson’s disease and multisystem atrophy [12]. Indeed, our patient’s autopsy revealed no amyloid plaques and only rare neurofibrillary tangles in the cortex adjacent to the hippocampi. Furthermore, α-synuclein and tau are known to spread in the central nervous system from one nerve cell to another in a prion-like fashion [30]. Therefore, focal and whole brain radiotherapy or spinal irradiation may accelerate neurodegeneration in the brain or spinal cord causing progressive delayed radiation-induced encephalopathy or myelopathy.

There are currently diagnostic markers of α-synucleinopathy for patients with subclinical disease. Phosphorylated α-synuclein can be identified in peripheral nerve endings from skin punch biopsy and decreased dopamine levels can be observed in the stratum by DaTscan [31, 32]. Furthermore, certain forms of α-synuclein aggregates can be identified in the cerebrospinal fluid [33]. Therefore, elderly patients with glioblastoma may benefit from these diagnostic studies and, when α-synucleinopathy is diagnosed, involved-field radiation may be administered with a more conformal plan to the tumor while avoiding subcortical regions that are at risk of damage. Alternatively, elderly glioblastoma patients could forgo radiation and be treated with temozolomide alone [34]. Similarly, for those at risk for Alzheimer’s disease, phosphorylated tau and β-amyloid can be detected in the cerebrospinal fluid [35] and patients with high levels of these misfolded proteins may need an alternative treatment plan. Unfortunately, there is no diagnostic study for TDP-43, FUS or Trf2 in the clinic. However, future use of any of the above phosphorylated or misfolded proteins, either individually or in combination, may help identify patients at risk for accelerated encephalopathy or myelopathy from radiation and therefore prompt the development of new treatment approaches.

In summary, we present a patient who developed rapid neurocognitive deterioration after treatment for a glioblastoma in the right temporal lobe. Postmortem examination of his brain revealed the presence of neuronal Lewy bodies. The presence of α-synucleinopathy could be a negative outcome modifier in glioblastoma patients and an alternative treatment strategy may be needed to preserve neurological function.

## Data Availability

Not applicable.

## Abbreviations

53BP1: P53 binding protein 1
ATM: Ataxia telangiectasia mutated
ATRX: α-Thalassemia mental retardation syndrome
cGy: CentiGray
CT: Computed tomography
DaTscan: Dopamine Transporter scan
dL: Deciliter
DNA: Deoxyribonucleic acid
FLAIR: Fluid-attenuated inversion recovery
FUS: Fused in sarcoma
γH2Ax: Gamma H2A histone family member X
GFAP: Glial fibrillary acidic protein
HSV: Herpes simplex virus
IDH: Isocitrate dehydrogenase
IU: International unit
Ki-67: Marker of proliferation 67
L: Liter
mg: Milligram
MP-RAGE: Magnetization-prepared rapid gradient-echo
MRI: Magnetic resonance imaging
Olig-2: Oligodendrocyte transcription factor-2
PARP: Poly-ADP-ribose polymerase
PCR: Polymerase chain reaction
PTV: Planned treatment volume
TDP-43: Transactive response DNA binding protein 43 kDa
Trf2: Telomeric repeat-binding factor 2
μm: Micron
VMAT: Volumetric modulated arc therapy
VZV: Varicella-zoster virus
